# Surgical resection of a giant retroperitoneal dedifferentiated liposarcoma: a case report

**DOI:** 10.3389/fsurg.2025.1650969

**Published:** 2025-09-04

**Authors:** Shijie Zhong, Wei Yang, Zhangchao Li, Ruixue Li, Bimang Fu

**Affiliations:** ^1^ Yiling Hospital of Yichang, Affiliated Yiling Hospital of China Three Gorges University, Yichang, Hubei, China; ^2^ Yunnan St. John’s Hospital, Kunming, Yunnan, China; ^3^ First Ward of Hepatobiliary Pancreatic Surgery Department, The Second Affiliated Hospital of Kunming Medical University, Kunming, Yunnan, China; ^4^ Fourth Ward of Hepatobiliary Pancreatic Surgery Department, The Second Affiliated Hospital of Kunming Medical University, Kunming, Yunnan, China

**Keywords:** case report, retroperitoneal liposarcoma, dedifferentiated liposarcoma, multidisciplinary treatment, surgical resection

## Abstract

**Background:**

Liposarcoma (LPS) is a rare mesenchymal soft tissue sarcoma, and dedifferentiated liposarcoma (DDLPS) represents a clinically significant and aggressive subtype. Retroperitoneal DDLPS poses significant clinical challenges due to its insidious onset, large tumor size at the time of diagnosis, complex anatomical relationships, and high rates of recurrence and multi-organ involvement.

**Case presentation:**

We present the case of a 64-year-old male who presented with a progressively enlarging abdominal mass, fatigue, and reduced appetite persisting for over five years. Imaging studies and interdisciplinary evaluation revealed a massive retroperitoneal tumor with invasion into multiple adjacent organs. The patient underwent comprehensive surgical resection involving the tumor, left kidney, spleen, and distal pancreas, along with diaphragmatic repair. Histopathological analysis confirmed high-grade dedifferentiated liposarcoma. Postoperatively, the patient developed mild complications, including pneumothorax, which were effectively managed. Postoperative imaging and laboratory examinations demonstrated that the tumor was completely resected, with the majority of organ functions effectively preserved.

**Conclusion:**

This case highlights the complexity of treating a large and multi-organ-involving retroperitoneal dedifferentiated liposarcoma. Radical surgical resection remains the most effective treatment approach, but while achieving complete resection, it is also necessary to take into account the maximum protection of the patient's vital organ functions.

## 1 Introduction

Liposarcoma (LPS) is a rare mesenchymal soft tissue sarcoma that originates from the adipocyte lineage and is generally believed to arise from adipocytes in soft tissues (1). LPS accounts for approximately 13%–20% of all soft tissue sarcomas and is one of the most common types of soft tissue sarcoma worldwide (2). According to the 2020 World Health Organization (WHO) classification criteria for LPS, its subtypes include atypical lipomatous tumor (ALT)/well-differentiated liposarcoma (WDLPS), dedifferentiated liposarcoma (DDLPS), myxoid liposarcoma (MLPS), pleomorphic liposarcoma (PLPS), and myxoid pleomorphic liposarcoma (MPLPS) (3). This type of tumor is frequently diagnosed at an advanced stage due to its indolent growth pattern and nonspecific early symptoms.

Among all subtypes of LPS, DDLPS holds significant clinical importance, accounting for approximately 20% of all LPS (4). Its clinical manifestations are characterized by significant local invasiveness and a high recurrence rate, and it is more frequently found in the retroperitoneum (5). Due to the strong extensibility of the retroperitoneal space, DDLPS lacks specific clinical manifestations in the early stage, and patients may only present with mild abdominal distension or discomfort at the initial stage (6). It is not until the tumor compresses or invades adjacent organs that obvious clinical symptoms occur (7). Therefore, many patients are diagnosed at an advanced stage with large tumor volumes, significantly increasing the difficulty of surgical treatment.

In clinical diagnosis, early LPS is often misdiagnosed due to its similar imaging manifestations to benign lipomas. Although imaging examinations such as CT and MRI are helpful in assessing the extent of the tumor and its relationship with adjacent structures, a retrospective analysis of 291 cases by Morosi indicates that, except for WDLPS, there are currently no clear radiological criteria to accurately distinguish all types of retroperitoneal sarcomas (8). Consequently, an accurate diagnosis typically necessitates a combination of imaging studies and histopathological evaluation (9).

DDLPS, due to its anatomical location and inherent biological features, frequently infiltrates neighboring vital organs, consequently elevating the complexity of surgical resection (10). During clinical treatment, multi-organ resection is often necessary to achieve complete tumor removal. The case described in this report features a large tumor volume, recurrence, multi-organ resection, and multidisciplinary decision-making, providing important reference value for the clinical management of complex liposarcoma and the multidisciplinary collaboration model. This case report is written in accordance with the SCARE 2025 (11) statements. For detailed content, please refer to Supplementary Materials.

## 2 Case presentation

### 2.1 Patient information and chief complaint

The patient is a 64-year-old male. He has no history of smoking or alcohol consumption and denies a family history of genetic disorders. The patient presented to the clinic primarily due to a progressively enlarging abdominal mass associated with fatigue and decreased appetite for more than five years. There was no notable weight loss, melena, or hematuria.

### 2.2 Past medical history and medication history

Five years ago, he was admitted to a local hospital due to gastrointestinal bleeding and incidentally discovered a renal mass. After discharge, he self-administered traditional Chinese medicine for treatment, but the specific formula is unknown. He has not undergone regular followup since then. The patient denies a history of diabetes, hypertension, or cardiovascular and cerebrovascular diseases. He also denies any previous surgical history.

### 2.3 Disease course review and timeline

In 2020, he was first admitted to a local hospital where an abdominal mass was detected during a physical examination, but no further treatment was pursued. At the end of 2022, an imaging follow-up at the local hospital indicated an increase in tumor size, and surgery was recommended. However, the patient and his family refused and continued with conservative treatment using traditional Chinese medicine. In May 2024, due to the increasing size of the abdominal mass, he underwent a biopsy at a hospital in Guiyang, Guizhou Province, China.

The results of the puncture biopsy pathology are as follows (Supplementary Materials):

1. CD34 is diffusely positive, suggesting a mesenchymal tumor.
2. This histological examination rules out neurogenic and myogenic tumors, but does not rule out well-differentiated liposarcoma.

The immunohistochemical results are as follows: Vimentin (+), CD34 (+),Bcl2 (+), CD99 (−), SATB2 (−), β-catenin (−), SMA (−), MSA (−), Desmin (−)CD117(−), DOG-1(−), S-100(−), CK(−), Ki67 (approximately 3%+), STAT-6(−), CDK4(+), MDM2(−). The patient refused further treatment due to personal reasons. On March 15, 2025, the patient was transferred to our hospital due to a significant increase in the abdominal mass.

### 2.4 Physical examination

**Figure 1:**
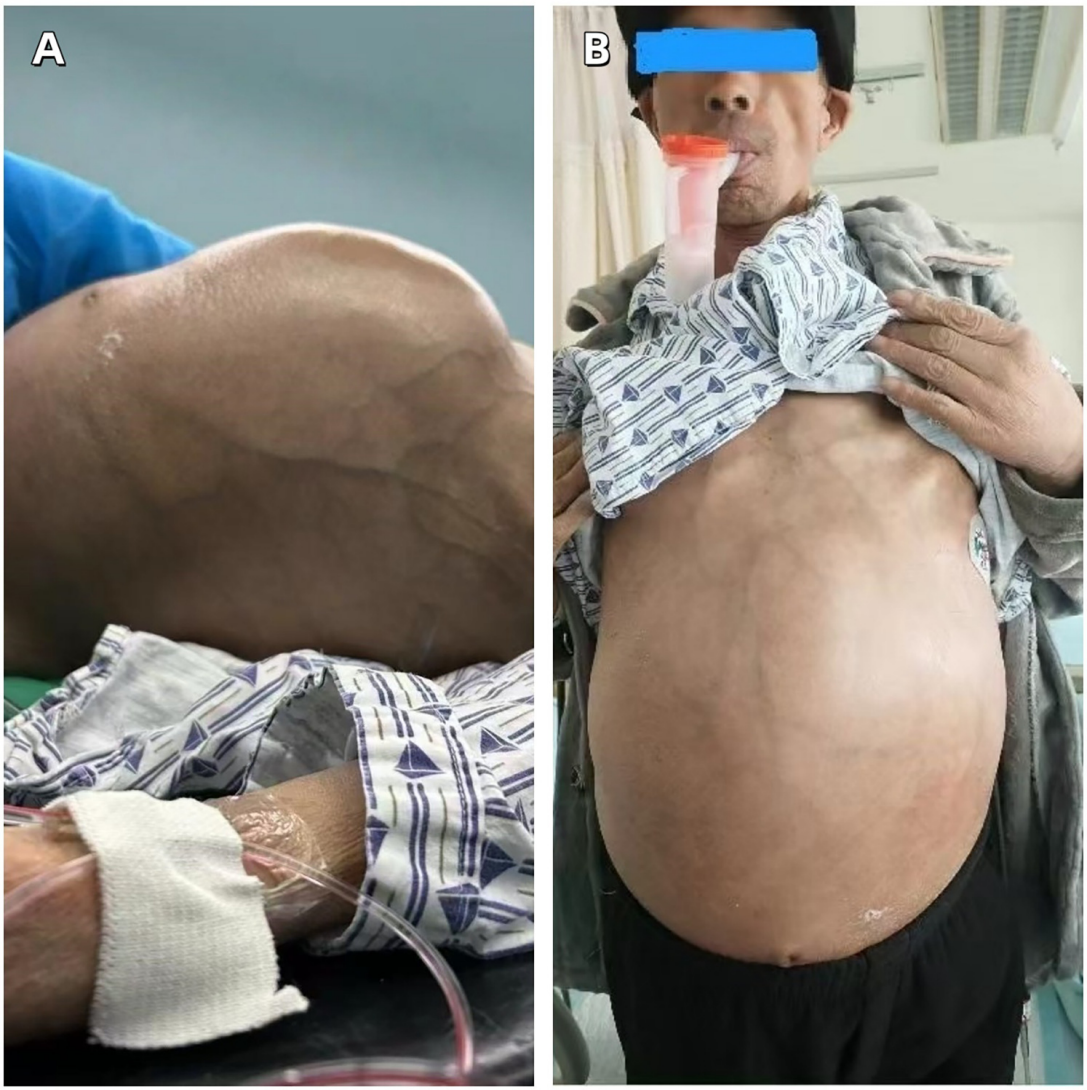
**(A)** A massive abdominal mass in the supine position. **(B)** A massive abdominal mass in the upright position.

On admission, the patient's pulse was 112 beats per minute, respiration 30 times per minute, and blood pressure 106/75 mmHg. The abdomen was distended. A large abdominal mass could be palpated in both the supine and standing positions (Figures 1A,B), and it exhibited a medium consistency. Varicosities of the abdominal wall veins were evident. Abdominal respiration was the primary mode of breathing, and no signs of gastric or intestinal patterns or peristaltic waves were detected. Mild abdominal tenderness was noted, without rebound tenderness, while muscular tension of the abdominal wall was present. Enlargement of the liver and spleen was not palpable below the costal margin. The upper border of the hepatic dullness was located at the fifth intercostal space on the right side. The fluid thrill test was negative, as was the shifting dullness test. The bowel sound frequency was 4 times per minute. No percussion pain was elicited over the hepatic and renal regions. Murphy's sign was negative. There was no tenderness at the costal margin point, upper ureteral point, middle ureteral point, costovertebral angle point, or costolumbar point. Moreover, no vascular murmurs were auscultated.

### 2.5 Laboratory and imaging findings

**Figure 2:**
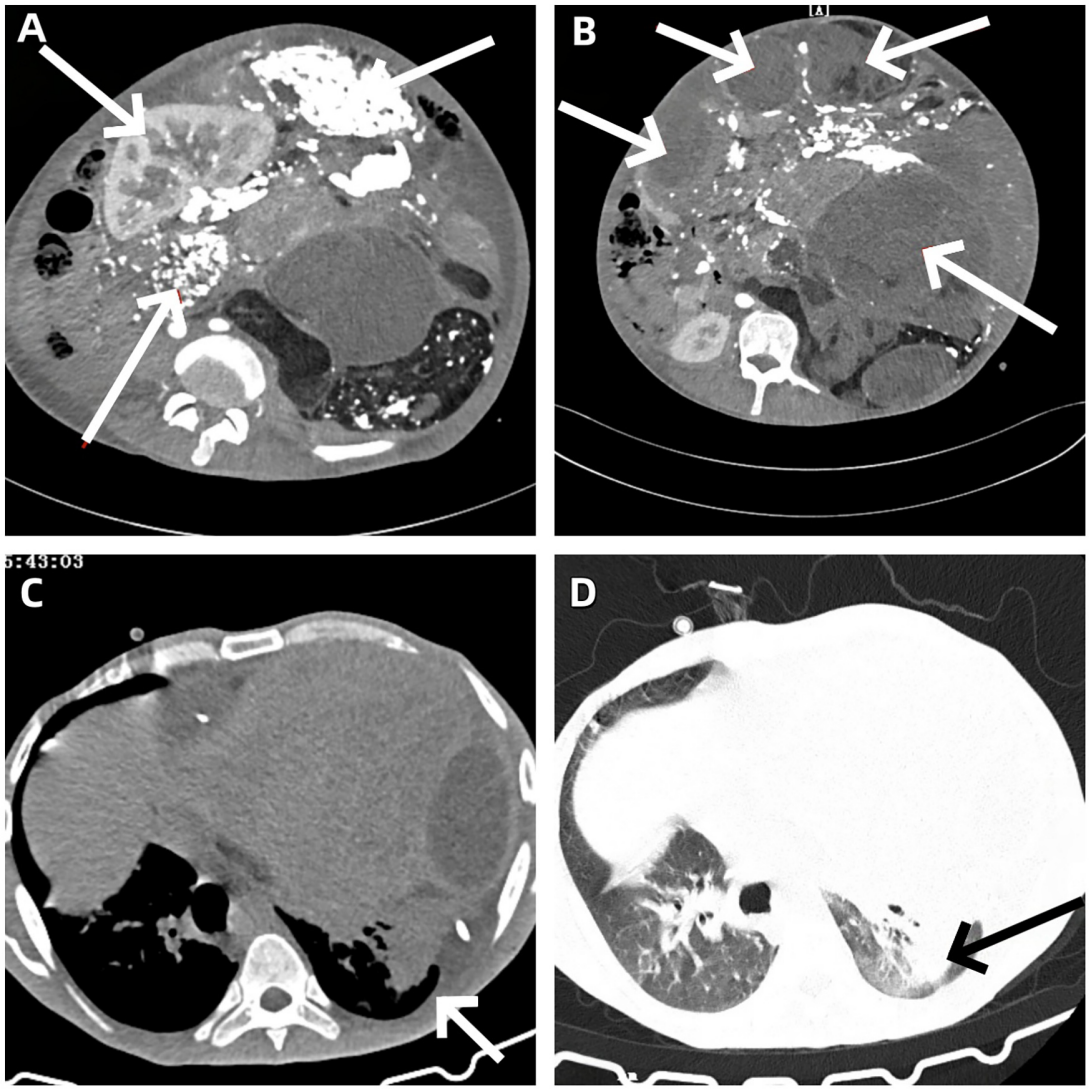
**(A)** A large heterogeneous-density mass is observed in the abdominal cavity, causing compression and displacement of the left kidney to the right abdominal region. **(B)** A large heterogeneous-density mass lesion in the pelvic cavity, with evidence of displacement and infiltration of adjacent organs. **(C)** Presence of left-sided pleural effusion. **(D)** Partial atelectasis of the left lung.

The blood test results showed an elevated white blood cell count, accompanied by mild anemia and a decreased albumin level. The remaining routine laboratory test results are detailed in Table 1. The plain chest CT combined with abdominal enhanced CT indicated the presence of a large mixed-density mass in the thoracic, abdominal and pelvic cavities (Figures 2A,B), which exerted compression and invasion on the surrounding organs, and was accompanied by left pleural effusion (Figure 2C), left lung atelectasis (Figure 2D), and pelvic effusion. The echocardiogram showed a small amount of pericardial effusion; abdominal ultrasound also indicated effusion in both the abdominal and thoracic cavities. The patient did not undergo PET-CT examination.

**Table 1:** Crucial laboratory tests before and after surgery.

Variables	HGB(g/L)	PLT(10^9/L)	GLU(mmol/L)	CRP(mg/L)	ALB(g/L)	ALT(U/L)	AST(U/L)	CREA(umol/L)	K^+^(mmol/L)	Na^+^(mmol/L)
Reference Range	130–175	125–350	3.33–6.11	<8	40–55	<41	<37	44–97	3.5–5.5	135–145
Preoperative	90↓	220	4.65	55↑	23.1↓	9.2	17.0	39↓	4.2	137.4
Two weeks postoperative	72↓	423↑	9.14↑	25.5↑	32.8↓	14.9	18.0	28↓	3.81	120.2↓

HGB, hemoglobin; PLT, platelet count; GLU, glucose; CRP, C-reactive protein; ALB, albumin; ALT, alanine aminotransferase; AST, aspartate aminotransferase; CREA, creatinine; K, potassium; Na, sodium.

### 2.6 Diagnosis and multidisciplinary evaluation

After a comprehensive analysis of the patient's imaging data, previous pathological results and clinical manifestations by the multidisciplinary team (MDT), a preliminary diagnosis of a huge DDLPS in the retroperitoneum was made. The tumor has invaded the left kidney, spleen, body and tail of the pancreas, and the diaphragm, which is a highly aggressive and recurrent soft tissue sarcoma. Due to the large tumor volume, it has continuously compressed and infiltrated the organs, blood vessels and lymphatic system in the abdominal, thoracic and pericardial cavities, resulting in obstruction of venous and lymphatic return, and subsequently causing ascites, pleural effusion and pericardial effusion. The increased volume load has also led to an increase in the patient's heart rate. These pathological and physiological changes have further exacerbated the patient's abdominal pain and distension symptoms after admission, and the condition has been progressively worsening. Given the locally aggressive growth characteristics of the tumor and the patient's aggravated clinical symptoms and significantly decreased quality of life, the multidisciplinary team unanimously believes that surgical intervention is a necessary treatment measure to improve the patient's quality of life.

## 3 Surgical procedure

### 3.1 Surgical findings and procedures

Following comprehensive communication with the patient and his family members, surgical intervention was implemented at our hospital on April 1, 2025. A midline incision of approximately 45 cm was made in the abdomen, and the skin, subcutaneous tissue, and peritoneum were successively incised to enter the abdominal cavity. During the operation, it was found that there was a small amount of light yellow fluid in the abdominal cavity, about 200 ml in total, which was removed with an aspirator. A huge tumor was visible in the abdominal cavity, enveloping and pushing the left kidney and spleen to the right side, invading the left kidney, spleen, and diaphragm, making it difficult to separate. The intestinal tubes were gathered on the right side, with no obvious adhesions and no invasion by the tumor.

**Figure 3:**
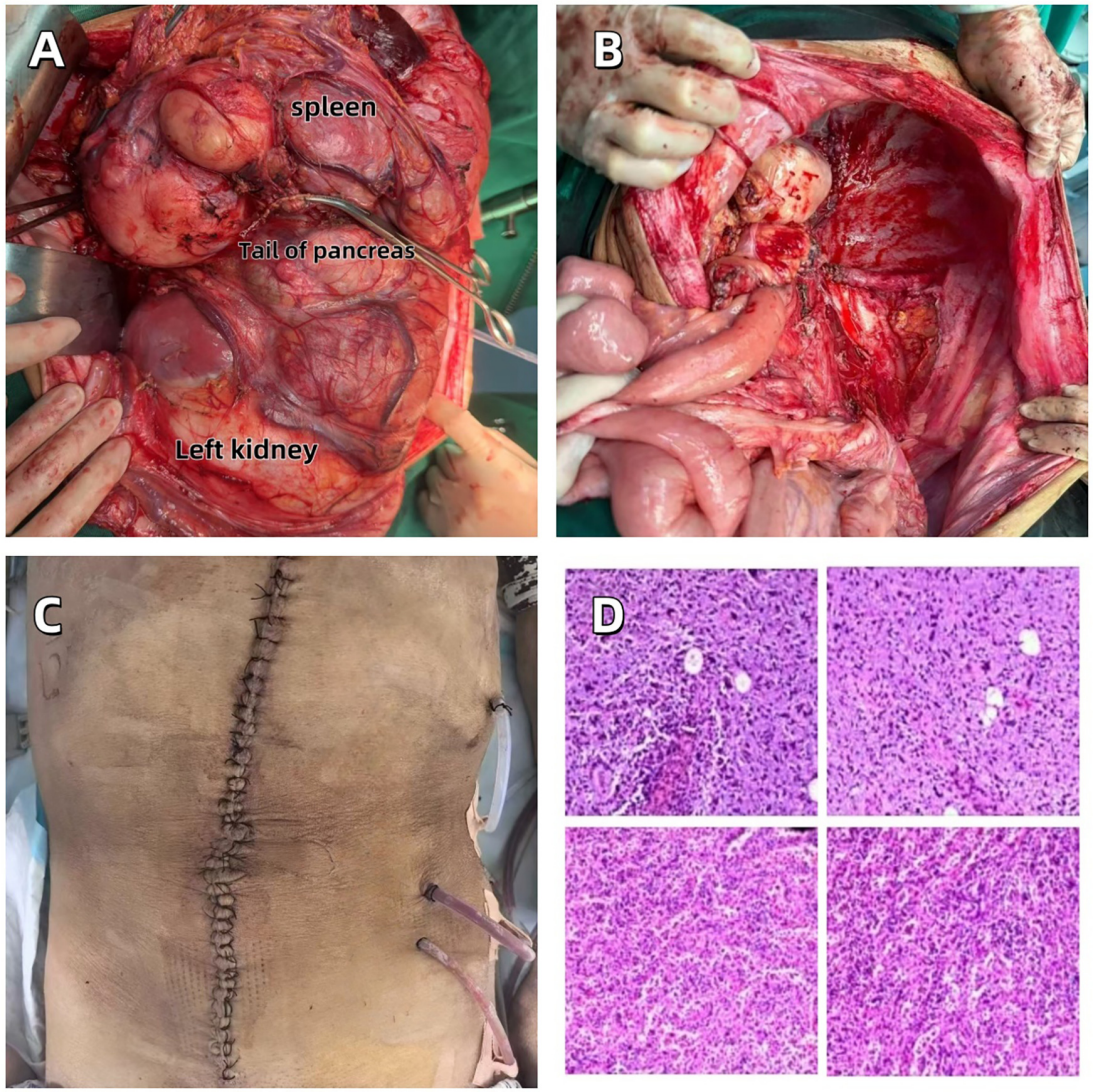
**(A)** Completely resected huge abdominal tumor. **(B)** The abdominal surgical field after tumor resection. **(C)** Abdominal incision in the postoperative recovery period. **(D)** Pathological section of well-differentiated dedifferentiated liposarcoma.

The patient underwent a comprehensive surgical procedure involving abdominal tumor resection, total splenectomy, left nephrectomy, distal pancreatectomy, and diaphragmatic repair. The tumor was completely resected and measured 42.0 cm × 36.0 cm × 18.0 cm, with an intact fibrous capsule observed macroscopically (Figures 3A,B). Postoperative imaging confirmed the absence of the previously noted large abdominal mass (Figure 3C).

The postoperative pathological diagnosis results are as follows (Supplementary Materials):

1. Considered to be liposarcoma;
2. A small amount of tumor infiltration was observed in the perirenal adipose tissue;
3. The tumor was found to involve the capsule of the spleen.

After consultation with experts from Jiangsu Provincial People's Hospital, China, the final diagnosis was high-grade dedifferentiated liposarcoma (Figure 3D).

The immunohistochemical test results are as follows: Tumor cells (−3): CD34 (+), SMA (−), S-100 (−), CD68 (a few +), Ki-67 (+, approximately 10%), CDK4 (−), MDM2 (scattered +), ALK (−); (−7): TFE-3 (−), CD68 (scattered +), CD163 (partially +), S-100 (−), PAX-8 (−), CAIX (−), CK7 (−), CgA (−), Syn (−); (−21): CgA (−), Syn (−).

**Figure 4:**
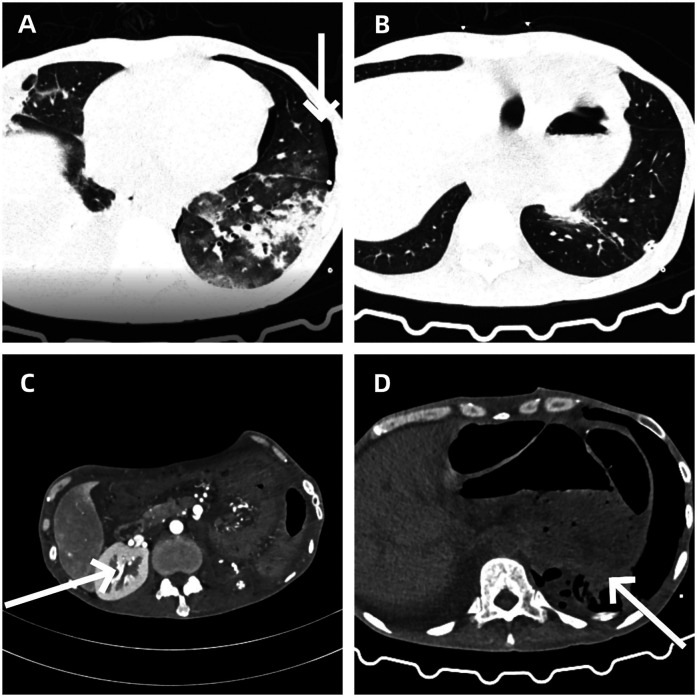
**(A)** Left-sided pneumothorax as a postoperative complication. **(B)** Gradual resolution of pneumothorax following closed thoracic drainage. **(C)** Abdominal contrast-enhanced CT confirms complete resection of the tumor. **(D)** The resolution of the subphrenic space-occupying lesion.

Two weeks after the operation, the patient underwent a blood routine test and related biochemical index tests (see Table 1). The results showed mild hyperglycemia and a decrease in hemoglobin levels, while no significant abnormalities were found in liver and kidney functions. The increase in blood sugar might be related to the systemic stress response after the operation, and the decrease in hemoglobin levels was consistent with the amount of blood loss during the operation. In the early postoperative follow-up of chest CT, the patient was found to have pneumothorax (Figure 4A). After closed thoracic drainage treatment, subsequent CT examinations indicated that the gas was gradually absorbed (Figure 4B). Abdominal enhanced CT confirmed that the tumor had been completely resected (Figure 4C), and the chest CT also showed that the space-occupying lesion under the diaphragm had disappeared (Figure 4D). The patient was discharged smoothly on April 20, 2025, without any other postoperative complications.

## 4 Discussion

This case fully demonstrates that through the close collaboration of a multidisciplinary team, complete resection of a large and anatomically complex retroperitoneal DDLPS is still achievable. During the operation, the key and challenge lies in ensuring complete tumor resection while maximizing the protection of the functions of surrounding organs. It is worth emphasizing that after tumor resection, the original compression on the internal organs was relieved, and the patient's clinical symptoms were significantly alleviated. Throughout the operation, the surgical team constantly balanced and adjusted between “thorough tumor resection” and “minimizing damage to important organs and maintaining the patient's physiological stability”, which is the core challenge in the surgical treatment of complex retroperitoneal tumors. Overall, this case provides valuable clinical experience and practical reference for surgical decision-making and implementation in similar complex situations.

Given the rarity of DDLPS and the intricacies of its biological behavior, accurate diagnosis poses a substantial challenge. This often leads to misdiagnosis or treatment delays, thereby forfeiting the optimal intervention window (1). In this case, the patient presented with progressive abdominal distension. Abdominal contrast-enhanced CT revealed multiple large masses, initially suggesting multiple malignant tumors. Although imaging studies can delineate the tumor's extent, the definitive confirmation of the subtype still hinges on multiple-site biopsies, histopathological examination, and immunohistochemical analysis. Eventually, the patient was diagnosed with DDLPS. However, due to the patient's financial constraints, PET-CT was not performed, potentially impeding the assessment of distant metastases.

Currently, surgical resection remains the most effective treatment modality for retroperitoneal DDLPS. Nevertheless, as the tumor frequently invades multiple organs, surgery often necessitates the combined resection of multiple organs. This not only escalates the risk of postoperative complications but also imposes more stringent demands on postoperative management (12). Surgeons should ensure tumor-free resection margins while endeavoring to preserve normal organ function to maintain the patient's physiological equilibrium and quality of life (13). In this case, via a midline abdominal incision, the abdominal tumor, spleen, left kidney, pancreatic body and tail, and a portion of the diaphragm were completely excised, achieving R0 resection. Postoperative pathology confirmed tumor involvement of the perirenal fat and the splenic capsule, further attesting to the thoroughness of the surgical resection. Systemic review studies have indicated that R0 resection is closely associated with improved patient prognosis (14). Although the patient experienced complications such as pneumothorax after surgery, these were within a manageable range. However, the literature also indicates that even with R0 resection margins, recurrence remains difficult to entirely prevent (15–17). This may be intricately linked to the degree of tumor dedifferentiation; the lower the degree of differentiation, the higher the recurrence risk and the poorer the prognosis (18). Consequently, surgery alone is insufficient to fully address the high recurrence rate of DDLPS, and a comprehensive treatment approach is required to optimize patient outcomes.

Regarding the role of chemotherapy in DDLPS, existing research has demonstrated its limited efficacy (19). However, individual case reports suggest that in some patients, neoadjuvant chemotherapy has led to a significant reduction in tumor volume, ultimately enabling radical resection. This implies that under specific circumstances, the combination of chemotherapy and surgery may confer clinical benefits (20). Nevertheless, such cases are infrequent and do not warrant routine recommendation. Emerging evidence suggests that trabectedin, as a second-line chemotherapeutic agent, demonstrates promising efficacy in the treatment of liposarcoma. By modulating the tumor microenvironment and extracellular matrix, it offers a viable therapeutic option for patients with refractory or recurrent disease (21). The combination of radiotherapy and surgery in the treatment of DDLPS has been extensively reported, encompassing preoperative, intraoperative, and postoperative modalities. Preoperative radiotherapy is the primary approach for most patients. For those with a low preoperative recurrence risk assessment but unfavorable pathological findings during surgery, postoperative radiotherapy serves a complementary purpose (3). Thus, the combination of surgery and radiotherapy may represent an ideal treatment strategy for DDLPS.

Among other treatment modalities, Brigimadlin (an MDM2 inhibitor) has exhibited potential antitumor activity in DDLPS/WDLPS (22). In DDLPS, CDK4 amplification occurs at a rate as high as 90%, suggesting its potential as a viable therapeutic target; however, the clinical efficacy of targeting CDK4 remains to be further investigated (23). Retrospective studies have also suggested that anlotinib, a multi-target vascular endothelial growth factor receptor inhibitor, may possess certain therapeutic efficacy for metastatic or recurrent DDLPS (20). Additionally, immunotherapy, radiofrequency ablation, CAR-T cell therapy, and TCR-T cell therapy offer alternative treatment options for inoperable patients (3).

The limitations of this study include the omission of FDG PET-CT, which may impact the assessment of distant metastases and the determination of long-term recurrence risk. Moreover, the dearth of long-term follow-up data precludes a comprehensive evaluation of the patient's long-term prognosis. In the future, the MDT collaborative mechanism should be further promoted, and the comprehensive treatment strategy centered on surgery should be fortified to enhance the clinical outcomes of patients with complex DDLPS.

### 4.1 Key clinical message

Successful management of giant dedifferentiated retroperitoneal liposarcoma necessitates a multidisciplinary approach and radical surgical intervention to achieve complete tumor resection while maximizing preservation of adjacent organs. Early diagnosis and individualized surgical planning play a critical role in improving clinical outcomes for patients with complex soft tissue sarcomas.

## Data Availability

The original contributions presented in the study are included in the article/Supplementary Material, further inquiries can be directed to the corresponding authors.
