# Supplementary material for: Surgical resection of a giant retroperitoneal dedifferentiated liposarcoma: a case report
Source: Front Surg. 2025 Sep 4;12:1650969. doi: 10.3389/fsurg.2025.1650969 (PMC12446831; doi:10.3389/fsurg.2025.1650969)
Supplement: Supplementary file 1 [file Datasheet1.pdf]

SCARE Guideline Checklist 2025			
**Topic**	**Item**	**Description**	**Page number**
**Title**	1	- The words ‘case report’ should appear in the title.<br>- The title should be concise and highlight the area of focus (e.g. presentation, patient population, diagnosis, surgical intervention, or outcome).	01
**Key Words**	2	- Include three to six keywords that identify what is covered in the case report (e.g. patient population, diagnosis or surgical intervention).<br>- Include ‘case report’ as one of the keywords.	01
**Highlights**	3	- Include three to five bullet points that capture the novel findings of the report.<br>- These should focus on providing a brief background to the report. Include the key results, their clinical relevance, and any validation performed.	01
**Abstract**	4a	**Structure**<br>- Provide a structured abstract that includes the following headings: (1) introduction and importance, (2) presentation of case, (3) clinical discussion, and (4) conclusion.	01
	4b	**Introduction and Importance**<br>- Describe what is known currently on this topic, what is important, unique or educational about the case, and what this adds to the surgical literature.	01-02
	4c	**Presentation of Case**<br>- Detail the presenting complaint(s), clinical and demographic details, and the patient’s main ideas, concerns, and expectations.<br>- Detail the clinical findings, investigations performed, main differentials, and subsequent diagnosis.<br>- Describe the rationale for choosing the intervention.<br>- Describe what was the outcome.	02-06
	4d	**Clinical Discussion**<br>- Discuss the clinical findings in relation to what is currently known.	06-07
	4e	**Conclusion**<br>- Describe the relevance and impact of the report.<br>- Detail the main take away lessons or potential implications for clinical practice (minimum of three).	01

**Artificial Intelligence (AI) (some journals may prefer this in the methods and/or acknowledgments section and it should also be declared in the cover letter)**	5	**Declaration of whether any AI was used in the research and manuscript development**<br><br>**If no, proceed to item 6.**<br>**If yes, proceed to item 5a**	
	5a	**Purpose and Scope of AI Use**<br>- Precisely state why AI was employed (e.g. development of research questions, language drafting, statistical analysis/summarisation, image annotation, etc).<br>- Was generative AI utilised and if so, how?<br>- Clarify the stage(s) of the reporting workflow affected (planning, writing, revisions, figure creation).<br>- Confirmation that the author(s) take responsibility for the integrity of the content affected/generated	
	5b	**AI Tool(s) and Configuration**<br>- Name each system (vendor, model, major version/date).<br>- State the date it was used<br>- Specify relevant parameters (e.g. prompt length, plug-ins, fine-tuning, temperature).<br>- Declare whether the tool operated locally on-premises, or via a cloud API and any integrations with other systems.	
	5c	**Data Inputs and Safeguards**<br>- Describe categories of data provided to the AI (patient text, de-identified images, literature abstracts).<br>- Confirm that all inputs were de-identified and compliant with GDPR/HIPAA.<br>- Note any institutional approvals or data-sharing agreements obtained.	
	5d	**Human Oversight and Verification**<br>- Identify the supervising author(s) who reviewed every AI output.<br>- Detail the process for fact-checking, clinical accuracy checks<br>- State whether any AI-generated text/figures were edited or discarded.<br>- Acknowledge the limitations of AI and its use	
	5e	**Bias, Ethics and Regulatory Compliance**<br>- Outline steps taken to detect and mitigate algorithmic bias (e.g.	

		cross-checking against under-represented populations).<br>- Affirm adherence to relevant ethical frameworks.<br>- Disclose any conflicts of interest or financial ties to AI vendors.	
	5f	**Reproducibility and Transparency**<br>- Provide the exact prompts or code snippets (as supplementary material if lengthy).<br>- Supply version-controlled logs or model cards where possible.<br>- if applicable, state repository, hyperlink or digital object identifier (DOI) where AI-generated artefacts can be accessed, enabling attempts at independent replication of the query/input.	
**Introduction**	6a	**Background**<br>- Describe the area of focus and the relevant background contextual knowledge.	01-02
	6b	**Rationale**<br>- Describe why the case is different to what is already known in the literature.<br>- Describe why it is important to report this case. Is the case rare or interesting for the specific healthcare setting, population or country?	02
	6c	**Guidelines and Literature**<br>- Give reference to relevant surgical literature and current standards of care, including any specific guidelines or reports (e.g. government, national, international).	02
**Guideline Citation**	7	- At the end of the introduction, include reference to the SCARE 2025 publication by stating: 'This case report has been reported in line with the SCARE checklist [include citation]'.	02
**Timeline**	8	- Summarise the sequence of events leading up to the patient's presentation.<br>- Report any delays from presentation to diagnosis and/or intervention.<br>- Use tables or figures to illustrate the timeline of events if needed.<br>- Use standardised units of time (mm:hh) and dates (dd/mm/yyyy).	02-03
**Patient Information**	9a	**Demographic Details**<br>- Include de-identified demographic information (e.g. age, sex, ethnicity, occupation).	02

		- Where relevant, include other useful information (e.g. body mass index, hand dominance, income, level of education, marital status).	
	9b	**Presentation**<br>- Describe the patient's presenting complaint(s).<br>- Include a collateral account of the history if relevant.<br>- Describe how the patient presented (e.g. self-presentation, ambulance or referred by family physician or other hospital clinicians).<br>- Describe where the patient presented (e.g. outpatient clinic, type of hospital, etv).	02
	9c	**Past Medical and Surgical History**<br>- Include any previous interactions (e.g. prior admissions to hospital), medical or surgical interventions, and relevant outcomes.	02
	9d	**Drug History and Allergies**<br>- Specify any acute, repeat, and discontinued medications.<br>- Specify any contraindications to re-starting regular medicines e.g. increased bleeding risk.<br>- Specify any allergies and/or adverse reactions.	02
	9e	**Family History**<br>- Include health information regarding first-degree relatives, specifying any inheritable conditions.<br>**Social History**<br>- Indicate any smoking, alcohol, and recreational drug use.<br>- Indicate the level of social independence, the presence of any carers, driving status, and type of accommodation.<br>**Review of Systems**<br>- Provide any other information outside of the focused history (e.g. headaches, blurred vision, palpitations, abdominal pain, joint pain).	02
**Clinical Findings**	10	- Describe the general and significant clinical findings based on initial inspection and physical examination.	02-03
**Diagnostic Assessment & Interpretation**	11a	**Diagnostics Assessment**<br>- Bedside (e.g. urinalysis, electrocardiography, echocardiography).<br>- Laboratory (e.g. biochemistry, haematology, immunology, microbiology, histopathology).<br>- Imaging (e.g. ultrasound, X-ray, CT/MRI/PET).<br>- Invasive (e.g. endoscopy, biopsy).	02-03

	11b	**Diagnostic Challenges**<br>- Where applicable, describe what was challenging about the diagnoses (e.g. access, financial, cultural).<br>- Describe how these challenges were overcome.	03-04
	11c	**Diagnostic Reasoning**<br>- Describe the differential diagnoses, why they were considered (e.g. given the initial presentation or after assessment and investigation), why and how they were excluded.	03-04
	11d	**Prognostic Characteristics**<br>- Include where applicable (e.g. tumour staging) and how this was performed.	03-04
**Intervention**	12a	**Pre-Operative Patient Optimisation**<br>- Lifestyle (e.g. weight loss).<br>- Medical (e.g. medication review, treating any relevant pre-existing medical concerns).<br>- Procedural (e.g. nil by mouth, enema).<br>- Other (e.g. psychological support).	04
	12b	**Surgical Interventions**<br>- Describe the type(s) of intervention(s) used (e.g. pharmacological, surgical, physiotherapy, psychological, preventative).<br>- Describe any concurrent treatments (e.g. antibiotics, analgesia, antiemetics, venous thromboembolism prophylaxis).<br>- Medical devices should have manufacturer and model specifically mentioned.	04-05
	12c	**Specific Details Regarding the Intervention**<br>- Describe the rationale behind the treatment offered, how it was performed and time to intervention.<br>- For surgery, include details on the intervention (e.g. anaesthesia, patient position, skin preparation used such as chlorhexidine or shaving, use of other relevant equipment, sutures, devices, surgical stage).<br>- For surgery, include any post-operative instructions (e.g. how long to keep an abdominal drain for, when to remove sutures or staples).<br>- The degree of novelty for a surgical technique/device should be mentioned (e.g. ‘first in human’).<br>- For pharmacological therapies, include information on the formulation, dosage, strength, route, and duration.	04-05

	12d	**Operator Details**<br>- Where applicable, include operator experience and position on the learning curve, prior relevant training, and specialisation (e.g. ‘junior trainee with 3 years of surgical specialty training’).<br>Setting of Intervention<br>- Specify the setting in which the intervention was performed (e.g. district general hospital, major trauma centre).<br>- Specify the level of experience that the centre has with performing the intervention.<br>- Specify whether the procedure was performed in collaboration with another speciality (e.g. a hybrid procedure).	N/A
	12e	**Deviation from Initial Management Plan**<br>- State if there were any changes in the planned intervention(s).<br>- Provide an explanation for these changes alongside the rationale (e.g. delays to intervention, a laparoscopic procedure converted to open due to operative difficulties).	N/A
**Follow-Up and Outcomes**	13a	**Specify Details Regarding the Follow-up**<br>- When (e.g. how long after discharge in months or years, frequency, maximum follow-up length at time of submission).<br>- Where (e.g. home via video consultation, primary care, secondary care).<br>- With whom (e.g. appointment with the original operating surgeon).<br>- How (e.g. telephone consultation, virtual or digital follow-up, clinical examination, blood tests, imaging).<br>- Any specific long-term surveillance requirements (e.g. imaging surveillance for endovascular aneurysm repair or clinical exam/ultrasound of regional lymph nodes for skin cancer).<br>- Any specific post-operative instructions (e.g. postoperative medications, targeted physiotherapy, psychological therapy).	N/A
	13b	**Intervention Adherence and Compliance**<br>- Where relevant, detail how well the patient adhered to and tolerated the advice provided (e.g. avoiding heavy lifting for abdominal surgery, or tolerance of chemotherapy and pharmacological agents).<br>- Explain how adherence and tolerance were measured.<br>- Explain whether these results will have an impact on the long-term applicability of the intervention in clinical practice.	N/A
	13c	**Outcomes**<br>- Expected versus attained clinical outcome as assessed by the clinician. Reference literature used to inform expected outcomes.<br>- When appropriate, include patient-reported measures (e.g.	06

		questionnaires including quality-of-life scales).<br>- Detail when the outcomes were recorded (e.g. at how many months or years post-operative).	
	13d	**Complications and Adverse Events**<br>- Precautionary measures taken to prevent complications (e.g. antibiotic or venous thromboembolism prophylaxis).<br>- All complications and adverse or unanticipated events should be described in detail and ideally categorised in accordance with the Clavien-Dindo Classification (e.g. blood loss, length of operative time, wound complications, re-exploration or revision surgery).<br>- If relevant, whether the complication was reported to the relevant national agency or pharmaceutical company.<br>- Specify the duration of time between completion of the intervention and discharge, and whether this was within the expected timeframe (if not, why not).<br>- Where applicable, the 30-day post-operative and long-term morbidity/mortality may need to be specified.<br>- Where applicable, specify whether any complications or adverse outcomes were discussed locally (e.g. during team or morbidity and mortality meetings).<br>- State if there were no complications or adverse outcomes.	06
**Discussion**	14a	**Summary of Results**<br>- Provide a clear summary of the key findings of the report.<br>- Provide a rationale for the conclusions drawn.	06-07
	14b	**Relevant Literature**<br>- Include a brief discussion of the relevant literature and, if appropriate, similar published cases.	07
	14c	**Future Implications**<br>- Describe the future implications for clinical practice and guidelines.	07
	14d	**Take Away Lessons**<br>- Outline the key clinical lessons from this case report.<br>- Discuss any differences in approach to diagnosis, investigation, or patient management which the authors might adopt in future cases, based on their experience of the current report.	07
**Strengths and Limitations**	15a	**Strengths**<br>- Describe the key strengths of the case.<br>- Detail any multidisciplinary or cross-specialty relevance.	07
	15b	**Weaknesses and Limitations**<br>- Describe the relevant weaknesses or limitations of the case.	07

		- If applicable, describe how these challenges were overcome.<br>- For novel techniques or devices, outline any contraindications and alternatives, potential risks and possible complications if applied to a larger population.	
**Patient Perspective**	16	- Where appropriate, the patient should be given the opportunity to share their perspective on the intervention(s) they received (e.g. sharing quotes from a consented and anonymised interview).	NA
**Informed Consent**	17	- The authors must provide evidence of consent, where applicable, and if requested by the journal.<br>- Consent should be provided for both the original intervention or procedure and publication of the current case report.<br>- State the method of consent at the end of the article (e.g. verbal, written, digital/virtual).<br>- If not provided by the patient, explain why (e.g. death of patient and consent provided by next of kin). If the patient or family members were untraceable, then document the tracing efforts undertaken.	**Supplementary Materials**
**Additional Information**	18	- Please state any author contributions, acknowledgements, conflicts of interest, sources of funding, and where required, institutional review board or ethical committee approval.<br>- Disclose whether the case has been presented at a conference or regional meeting.<br>- Disclose whether this case is under consideration at any other journal.	07-08
**Clinical Images and Videos**	19	- Where relevant and available, include clinical images to help demonstrate the case pre-, peri-, and post-intervention (e.g. radiological, histopathological, patient photographs, intra-operative images).<br>- Where relevant, ensure images are adequately annotated.<br>- Where relevant and available, a link (e.g. Google Drive, YouTube) to the narrated operative video can be included to highlight specific techniques or operative findings.<br>- Ensure all media files are appropriately captioned and indicate points of interest to allow for easy interpretation.	03-06
