# Supplementary material for: Surgical resection of a giant retroperitoneal dedifferentiated liposarcoma: a case report
Source: Front Surg. 2025 Sep 4;12:1650969. doi: 10.3389/fsurg.2025.1650969 (PMC12446831; doi:10.3389/fsurg.2025.1650969)
Supplement: Supplementary file 2 [file Datasheet2.pdf]

医学研究知情同意书

本人已清楚获悉，自己的医疗记录、影像资料以及相关临床信息将会被用于医学科研和出版（包括在学术期刊发表病例报告）。相关资料可包括（但不限于）病史、体格检查结果、影像学检查图片、术中和术后照片等。

本人已由研究人员详细告知，所有公开发表的内容都不会泄露我的姓名、身份证号等个人隐私信息，本人身份不会在任何公开发表的资料中被披露。

本人同意将我的病例信息用于医学科研与论文发表，且此同意为自愿签署，并已理解相关内容。

患者签名：杨光德

日期：2024年4月20日

（如患者本人无法签署，则法定监护人签名）

监护人签名：______________

与患者关系：______________

日期：____________________
