# Supplementary material for: Surgical resection of a giant retroperitoneal dedifferentiated liposarcoma: a case report
Source: Front Surg. 2025 Sep 4;12:1650969. doi: 10.3389/fsurg.2025.1650969 (PMC12446831; doi:10.3389/fsurg.2025.1650969)
Supplement: Supplementary file 3 [file Datasheet3.pdf]

贵州医科大学附属肿瘤医院

病理补充图文报告

病理号：[redacted]

姓名：[redacted] 性别：男 年龄：44 岁 住院号：[redacted]

院别：本院 科室 [redacted] 床号： 送检医师：[redacted]

临床诊断：1:肾恶性肿瘤 肾恶性肿瘤 接收日期 [redacted]

送检材料：腹腔占位

肉眼所见：
腹腔占位：灰白组织2条，长0.6cm-1.2cm，直径0.1cm （全）
蜡块总数：1个
HE浸染切片：1张

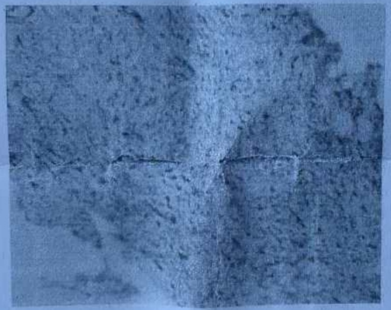

病理诊断：
第二次报告：
【腹腔占位】送检穿刺组织内见梭形细胞增生，CD34弥漫+，考虑间叶源性肿瘤，本次组化除外神经源性、肌源性肿瘤，考虑不除外高分化脂肪肉瘤，因活检标本组织少，待手术标本切除送检进一步明确或上传远程专家会诊协助诊断。
免疫组化结果显示：Vimentin(+),CD34(+),Bcl-2(+),CD99(－),SATB2(－),β-catenin(－),SMA(－),MSA(－),Desmin(－),CD117(－),DOG-1(－),S-100(－),CK(－),Ki67(+约3%),STAT-6(-),CDK4(+),MDM2(-)。

报告医师：[redacted] 审核医师：[redacted] 诊断日期：[redacted]

本报告仅对本次送检标本负责 如有不清之处请与病科室联系 联系电话：[redacted]
