# Supplementary material for: Surgical resection of a giant retroperitoneal dedifferentiated liposarcoma: a case report
Source: Front Surg. 2025 Sep 4;12:1650969. doi: 10.3389/fsurg.2025.1650969 (PMC12446831; doi:10.3389/fsurg.2025.1650969)
Supplement: Supplementary file 4 [file Datasheet4.pdf]

云南艾迪康医学检验所
YunNan ADICON Clinical Laboratories,Inc.

扫一扫查询报告

病理诊断报告

条形码: [illegible]

**姓 名:** [illegible]	**门诊/住院号:** [illegible]	**院方条码:** [illegible]	**联系电话:** [illegible]
**性 别:** 男	**科室/病区:** [illegible]	**送检医生:** [illegible]	**其他信息:** 7个工作日下午 3 点后取报告
**年 龄:** 45 岁	**送检组织:** 腹腔肿瘤	**临床诊断:** 腹腔肿瘤	
**床 号:** [redacted]	**病理号:** [redacted]	**送检单位:** 云南圣约翰医院	

【大体描述】:

核对送检标本、条形码与申请单一致，福尔马林固定标本。

（腹腔肿瘤）灰红色组织一块，结节样，大小 20.0cm*16.0cm*13.0cm，带骨，包膜完整，切面淡黄、灰红色。（蜡块 9 个）

【镜检所见】:

肿瘤细胞呈梭形，伴坏死，骨化，粘液样变性，部分区域见脂肪组织及脂母细胞。

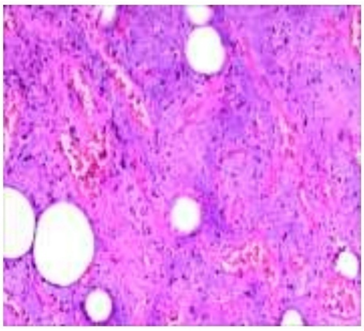

(图

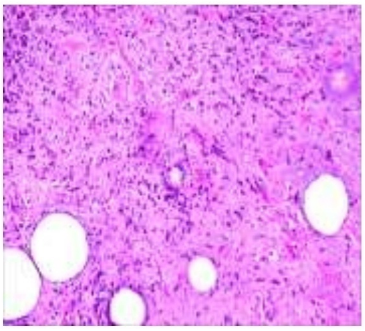

1)(图 2)

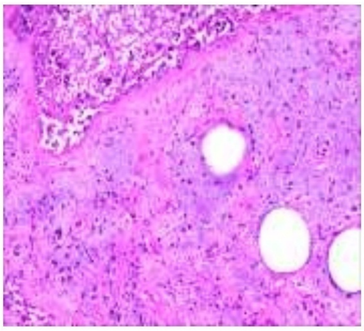

(图

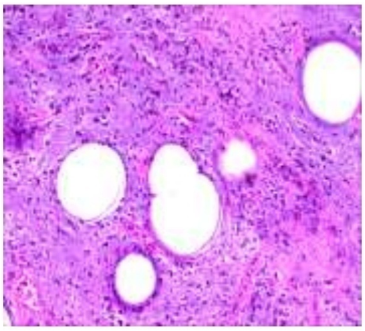

3)(图 4)

【病理诊断】:

（腹腔肿瘤）考虑脂肪肉瘤，待参照 [illegible] 号免疫组化结果确诊。

备注:

诊断医师: [illegible] **审核医师:** [illegible]

采集时间:[illegible] 接收时间:[illegible] 报告时间:[illegible] 报告编码[illegible] 主检实验室:云南艾迪康医学检验所本次检验结果仅对来样负责，如有疑问请在样本保存期内提出（样本保存期请参见艾迪康检测目录）。

报告无诊断医师、审核医师签字无效。如需借阅病理切片，请关注艾迪康医学检验服务号。

地址：[redacted] 网址：[illegible]

杭州艾迪康医学检验中心有限公司
HangZhou ADICON Clinical Laboratories Co.,Ltd

病理会诊咨询诊断报告

[illegible]

条形码：[illegible]

姓名：[illegible]	门诊/住院号：[illegible]	院方条码：[illegible]	联系电话：[illegible]
性别：男	科室/病区：[illegible]	送检医生：[illegible]	其他信息：\
年龄：45 岁	送检组织：	临床诊断：腹腔肿瘤	
床号：[illegible]	病理号：[illegible]	送检单位：云南圣约翰医院	

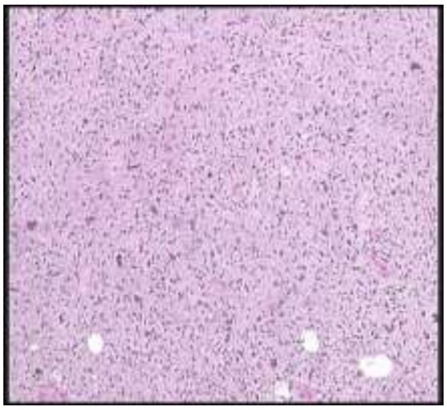
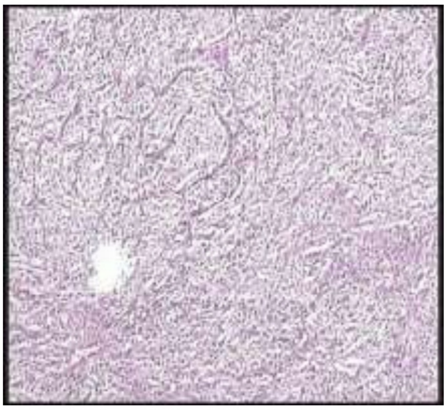

病理诊断：
送检单位：云南圣约翰医院；
送检材料：云南艾迪康(B2025007449--21)：蜡块*1，(B2025007449--20)：蜡块*1，(B2025007449--19)：蜡块*1，(B2025007449--18)：蜡块*1，(B2025007449--17)：蜡块*1，(B2025007449--16)：蜡块*1，(B2025007449--15)：蜡块*1，(B2025007449--14)：蜡块*1，(B2025007449--13)：蜡块*1，(B2025007449--12)：蜡块*1，(B2025007449--11)：蜡块*1，(B2025007449--10)：蜡块*1，(B2025007449--9)：蜡块*1，(B2025007449--8)：蜡块*1，(B2025007449--7)：蜡块*1，(B2025007449--6)：蜡块*1，(B2025007449--5)：蜡块*1，(B2025007449-4)：蜡块*1，(B2025007449--3)：蜡块*1，(B2025007449--2)：蜡块*1，(B2025007449--1)：蜡块*1，(B2025007449-1-21)：HE*21；
病理会诊意见：
（腹腔肿瘤）高级别去分化脂肪肉瘤。
免疫组化：肿瘤细胞（-3）CD34（+）， SMA（-）， S-100（-）， CD68（少量+）， Ki-67（+，约 10%）， CDK4（-），MDM2（散+）， ALK（-）。
（-7）TFE-3（-）， CD68（散+）， CD163（部分+）， S-100（-）， pax-8（-）， CAIX（-）， CK7（-），CgA（-），Syn（-）。
（-21）CgA（-）， Syn（-）。

备注：本例经江苏省人民医院[redacted]授会诊。
会诊资料局限，以上报告仅为所见切片的咨询意见，仅供原单位病理科参考。如治疗，请结合原单位临床资料综合分析。

杭州艾迪康医学检验中心有限公司
检测报告专用章
33010610421260

报告无诊断医师、审核医师、批准人签字无效；报告复印件无效。如需借阅病理切片，请携带就诊医院或送检医院盖有公章的借片单、借片人身份证及押金前来本中心办理。

地址：

邮编：

电话：

网址：

云南艾迪康医学检验所
YunNan ADICON Clinical Laboratories,Inc.

病理诊断报告

扫一扫查看报告

条形码:

姓　名: [illegible]　**门诊/住院号:** [illegible]　**院方条码:** [illegible]　**联系电话:** [redacted]

性　别: 男　**科室/病区:** [illegible]　**送检医生:** [illegible]　**其他信息:** 7个工作日下午 3 点后取报告

[redacted]

年　龄: 45　岁　**送检组织:** 腹腔肿瘤　**临床诊断:** 腹腔恶性肿瘤

床　号: [redacted]　**病理号:** [redacted]　**送检单位:** 云南圣约翰医院

【大体描述】:

核对送检标本、条形码与申请单一致，福尔马林固定标本。

腹腔肿瘤标本一个，大小 42.0cm*36.0cm*18.0cm，包膜完整，带肾组织一个，部分脾脏组织，脾脏组织与肿块包膜粘连，切面灰白、淡黄色，部分区域带粘液，部分区域呈半透明样，见一囊腔形成，组织中部分区域质硬如石，肾脏大小 10.0cm*6.0cm*4.0cm，剖开肾脏组织，肉眼未见明显异常，脾脏大小 11.0cm*10.0cm*3.0cm，各切面未见明显异常。（蜡块 21 个）

【镜检所见】:

肿瘤细胞呈梭形，伴坏死，骨化，粘液样变性，部分区域见脂肪组织及脂母细胞。

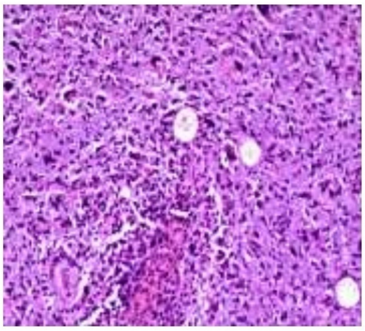

(图

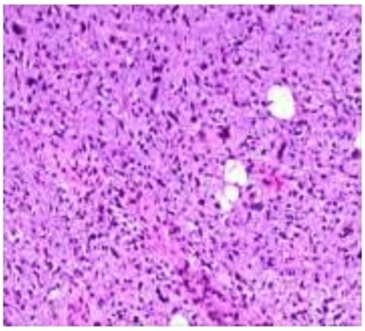

1)(图 2)

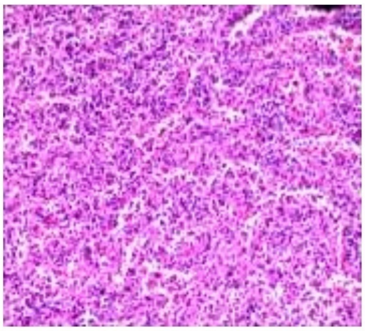

(图

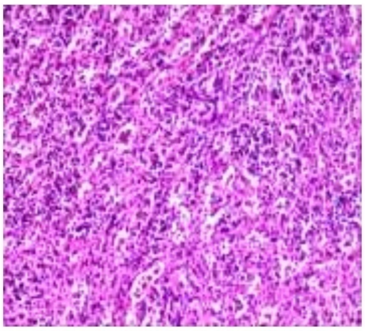

3)(图 4)

【病理诊断】:

（腹腔肿瘤+肾脏+脾脏）

1.考虑脂肪肉瘤，建议 3 号片加做免疫组化 14 项助诊；

2.肾脏周围脂肪组织中见少许肿瘤累及；

3.脾脏被膜见肿瘤累及。

备注:

诊断医师: [redacted] **审核医师:** [redacted]

采集时间: [redacted] 接收时间: [redacted] 报告时间: [redacted] 报告编码: [redacted] 主检实验室:云南艾迪康医学检验所本次检验结果仅对来样负责，如有疑问请在样本保存期内提出（样本保存期请参见艾迪康检测目录）。

报告无诊断医师、审核医师签字无效。如需借阅病理切片，请关注艾迪康医学检验服务号。

地址: [redacted] 网址: [redacted]

云南艾迪康医学检验所
YunNan ADICON Clinical Laboratories,Inc.
病理加做申请单

条形码: [redacted]

**姓　名:** [redacted]	**门诊/住院号:** [redacted]	**院方条码:** [redacted]	**病理号:** [redacted]
**性　别:** 男	**科室/病区:** [redacted]	**送检医生:** [redacted]	**其他信息:** 7个工作日下午 3 点后取报告
**年　龄:** 45　岁	**送检组织:** 腹腔肿瘤	**临床诊断:** 腹腔恶性肿瘤	
**床　号:** [redacted]	**联系电话:** [redacted]	**送检单位:** 云南圣约翰医院	

【申请内容】: 建议 3 号片加做免疫组化 14 项助诊;

【其他要求】: 若存在其他要求，请送检医生填写

【签字确认】:

若同意以上加做项目，请送检医生签名： 日期： 年 月 日

备注:

诊断医师: [redacted] **审核医师:** [redacted] **批 准 人:** [illegible]

采集时间 [redacted] 接收时间: [redacted] 报告时间:[redacted] 报告编码:[redacted] 主检实验室:云南艾迪康医学检验所本检测结果仅对来样负责，供临床参考。如有疑问请在样品保存期内提出（样品保存期请参见艾迪康检测目录）。

报告无诊断医师、审核医师、批准人签字无效；报告复印件无效。

如需借阅病理切片，请携带就诊医院或送检医院盖有公章的借片单、借片人身份证及押金前来本中心办理。

地址：[illegible] 邮编：[illegible] 客服热线：[illegible]

[illegible]
